# Supplementary material for: Valorization of Wheat Bran by Three Fungi Solid-State Fermentation: Physicochemical Properties, Antioxidant Activity and Flavor Characteristics
Source: Foods. 2022 Jun 13;11(12):1722. doi: 10.3390/foods11121722 (PMC9222537; doi:10.3390/foods11121722)
Supplement: Supplementary file 1 [file foods-11-01722-s001.zip › foods-1738228-supplementary.pdf]

Supporting Information

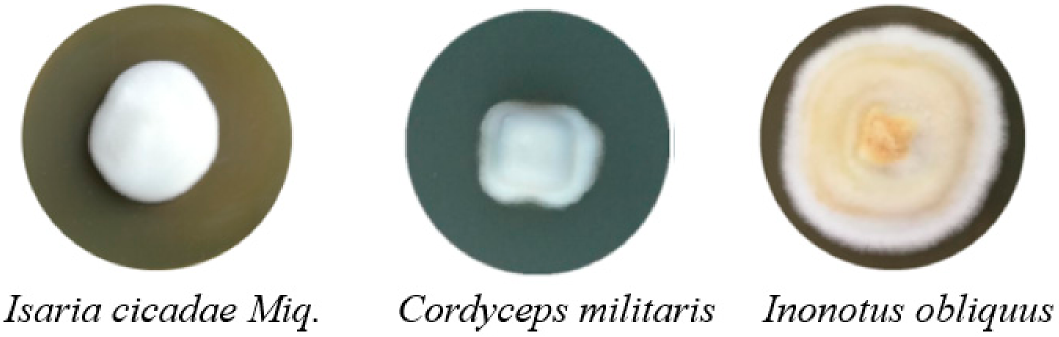

Figure S1. Colony photos of three medicinal fungi (*Isaria cicadae Miq.*; *Cordyceps militaris*; *Inonotus obliquus*)

Table S1. The chemical composition of the wheat bran powder

Composition	Content (%)
Starch	18.31±0.09
Protein	14.77±0.30
Fat	2.96±0.03
Water	12.28±0.09
Ash	5.74±0.06
IDF	42.19±0.17
SDF	3.75±0.05

Note: Data are expressed as mean ± standard deviation. IDF, insoluble dietary fiber; SDF, soluble dietary fiber.

Table S2. The relative molecular weight of soluble dietary fiber (SDF)

Molecular weight	US-SDF(%)	IC-SDF(%)	CM-SDF(%)	IO-SDF(%)
$>2\times10^6$ Da	38.43±0.21[c]	82.53±0.74[a]	49.60±0.27[b]	36.59±0.59[d]
2×10^5-2×10^6 Da	37.08±0.30[a]	13.95±0.44[d]	30.50±0.39[c]	31.76±0.23[b]
1×10^4-2×10^5 Da	17.73±0.48[a]	3.52±0.30[d]	7.15±0.26[c]	11.35±0.36[b]
$<1\times10^4$ Da	6.76±0.58[c]	0.00±0.00[d]	12.75±0.14[b]	20.30±0.71[a]

Note: Data are expressed as mean ± standard deviation. Different lowercase letters indicate significant differences at $p < 0.05$ in in terms of microorganisms. US-SDF, unfermented sterilized wheat bran soluble dietary fiber; IC-SDF, *Isaria cicadae Miq.* fermented wheat bran soluble dietary fiber; CM-SDF, *Cordyceps militaris* fermented wheat bran soluble dietary fiber; IO-SDF, *Inonotus obliquus* fermented wheat bran soluble dietary fiber.
